# Evidence on the Use of Mouthwash for the Control of Supragingival Biofilm and Its Potential Adverse Effects

**DOI:** 10.3390/antibiotics11060727

**Published:** 2022-05-28

**Authors:** Shoji Takenaka, Maki Sotozono, Naoto Ohkura, Yuichiro Noiri

**Affiliations:** Division of Cariology, Operative Dentistry and Endodontics, Faculty of Dentistry & Graduate School of Medical and Dental Sciences, Niigata University, Niigata 951-8514, Japan; sotozono@dent.niigata-u.ac.jp (M.S.); ohkura@dent.niigata-u.ac.jp (N.O.); noiri@dent.niigata-u.ac.jp (Y.N.)

**Keywords:** mouthwash, dental biofilm, gingivitis, cariostatic property, bacterial adhesion, microbiota, adverse reaction

## Abstract

Antimicrobial mouthwash improves supragingival biofilm control when used in conjunction with mechanical removal as part of an oral hygiene routine. Mouthwash is intended to suppress bacterial adhesion during biofilm formation processes and is not aimed at mature biofilms. The most common evidence-based effects of mouthwash on the subgingival biofilm include the inhibition of biofilm accumulation and its anti-gingivitis property, followed by its cariostatic activities. There has been no significant change in the strength of the evidence over the last decade. A strategy for biofilm control that relies on the elimination of bacteria may cause a variety of side effects. The exposure of mature oral biofilms to mouthwash is associated with several possible adverse reactions, such as the emergence of resistant strains, the effects of the residual structure, enhanced pathogenicity following retarded penetration, and ecological changes to the microbiota. These concerns require further elucidation. This review aims to reconfirm the intended effects of mouthwash on oral biofilm control by summarizing systematic reviews from the last decade and to discuss the limitations of mouthwash and potential adverse reactions to its use. In the future, the strategy for oral biofilm control may shift to reducing the biofilm by detaching it or modulating its quality, rather than eliminating it, to preserve the benefits of the normal resident oral microflora.

## 1. Introduction

**Figure 1:**
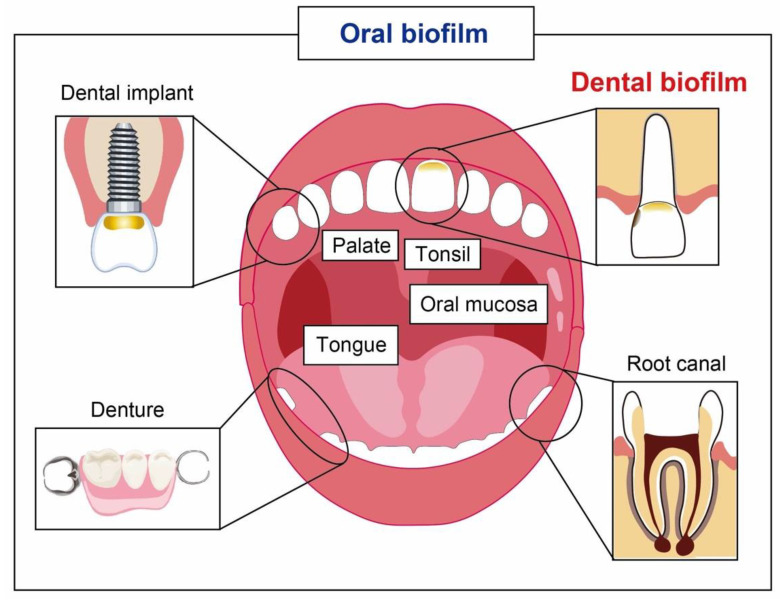
Oral biofilm and its niches.

More than 700 bacterial species or phylotypes, of which over 50% have not yet been cultivated, inhabit the biofilm that forms on the teeth, gingiva, tongue, oral mucosa, tonsil, palate, and dental materials [1,2]. A dental biofilm is a community of microorganisms found on the tooth surface (Figure 1) [3]. It is the primary etiology of oral infections and related disorders, such as dental caries [4,5], periodontal diseases [6,7], and root canal infections [8,9]. Dental biofilms have unique properties relative to the biofilms found in other parts of the human body. First, numerous and diverse microorganisms reside in the oral environment and coexist with the host. The relationship between the host and the biofilm formed by commensal bacteria is antagonistic, symbiotic, and mutualistic [10,11]. The commensal bacteria contribute to optimal tissue structure and function, as well as protecting the host from exogenous microorganisms by saturating niches [10]. Recent co-culture studies using a 3D organotypic reconstructed human gingiva model and a multi-species biofilm revealed that the commensal oral biofilm contributes to maintaining the homeostasis of human gingival barrier functions [12,13]. Thus, strategies to control the oral biofilm should involve preserving the benefits of the normal resident microflora.

Second, most biofilms can be removed using mechanical instruments, without requiring surgical intervention [14]. After removing the biofilm, the biological tissue will heal without saturing the wound site. The mechanical approach is therefore fundamental to the control of the oral biofilm.

The control of the supragingival biofilm is important for the prevention of periodontal diseases, as well as dental caries. The mechanical disruption and elimination of the dental biofilm can be effectively accomplished with the use of mechanical instruments, such as toothbrushes and dental floss [15]. However, it has been reported that mechanical control alone may be inadequate to prevent periodontal diseases due to limited time, limited use of interdental aids, inadequate patient skill, poor motivation, and lack of compliance [16,17]. In addition, the adequate cleaning of hard-to-reach areas and the gingival margin is difficult for older adults with physical or mental limitations, malpositioned or isolated teeth, fixed prostheses, or orthodontic appliances [18]. Furthermore, daily brushing is difficult for pregnant women and those with a strong vomiting reflex.

Various antimicrobial agents have been formulated into oral care products to supplement the effects of mechanical elimination [19,20,21]. Among them, mouthwash has demonstrated adjunctive efficacy with a high level of evidence. The American Dental Association (ADA)’s Council on Scientific Affairs has adopted specific guidelines to approve these chemotherapeutic agents [22]. The guidelines demand proof of plaque inhibitory and antiplaque activities based on double-blind randomized clinical trials for a minimum duration of 6 months. The most widely investigated antimicrobial mouthwashes are those containing chlorhexidine gluconate (CHG) [23,24,25], essential oils (EOs) [23,24,25,26], and cetylpyridinium chloride (CPC) [27]. The efficacies of mouthwashes containing povidone-iodine [28], delmopinol [29], hexetidine [30], polyhexanide [31], chlorine dioxide [31], sodium hypochlorite [32], chlorine dioxide [33], natural compounds [34], and hydrogen peroxide [35] have also been reported. Most mouthwashes reduce biofilm accumulation and gingival inflammation; however, the strength of the evidence supporting their effects varies.

The coronavirus disease pandemic has provided dental professionals with an opportunity to reconsider infection control during treatment. Mouthwash has been reconsidered for infection control in dental offices. The use of preprocedural mouth rinsing to reduce the contamination of the aerosols produced during dental procedures has been reported for the past 50 years [36]. Preprocedural mouth rinsing is a simple and effective way of reducing the number of microorganisms in dental aerosols [37].

This review aims to reconfirm the effects of mouthwash on biofilm control by summarizing systematic reviews within the last decade and discuss its limitations and potential adverse reactions, especially in relation to biofilm control strategies relying on bacterial elimination.

## 2. The Process of Biofilm Formation and Anti-Biofilm Strategy

**Figure 2:**
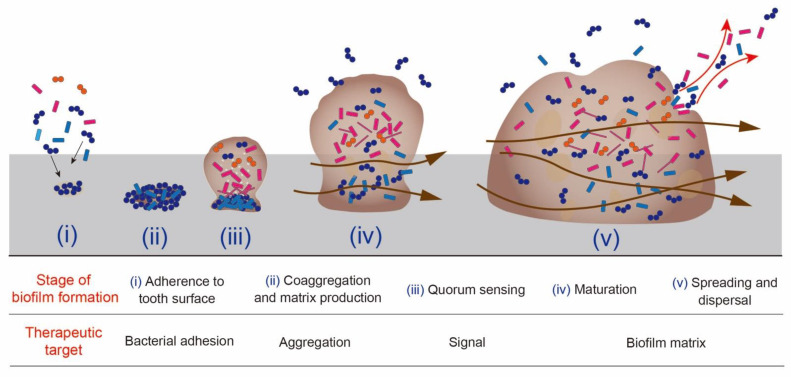
Biofilm formation and the therapeutic target for its control at each stage. The brown arrows indicate the channels that allow the transport of nutrients, waste products, and signaling molecules within the biofilm. The image is based on the process described by Costerton and Stewart in 2001 [38].

The process of biofilm formation can be summarized into the following five major stages: (i) adherence to tooth surface, (ii) coaggregation and matrix production, (iii) quorum sensing, (iv) maturation, (v) spreading and dispersal (Figure 2) [38]. Bacteria that settle on the adhesion interface (i) secrete extracellular polymeric substances that hold the cell aggregates together (ii). Cells grow through bacterial intercellular communication and initiate the formation of biofilm communities (iii). Within a mature biofilm population, cells express genetic and physiological heterogeneity in adapting to the local environmental conditions (iv). Some bacteria disperse and colonize other locations (v). This is the lifecycle of biofilms, and each stage proceeds in response to the surrounding environment [39,40]. Therefore, the therapeutic target for the control of biofilm changes with the development of the biofilm.

An anti-biofilm strategy involves a natural or induced process that leads to a reduction in the bacterial biomass through the alteration of the biofilm formation, integrity, and/or quality [41]. The currently available approaches to modulating biofilm formation include the inhibition of bacterial surface attachment and destabilization and/or the disruption of irreversibly attached mature biofilms. If an anti-biofilm agent is bactericidal, the agent should be very specifically targeted, otherwise its use could affect the composition of an established ecosystem and damage beneficial microbiota [41].

Currently, the onset and progress of dental caries and periodontitis are considered to be related to imbalanced microecology, also called dysbiosis [42,43]. Although the oral microbial community maintains a symbiotic relationship, external stimuli and accompanying environmental changes within the biofilm due to dietary sucrose uptake, poor oral hygiene, and salivary dysfunction cause the overgrowth of acidogenic and aciduric bacteria, resulting in an imbalance between the bacteria and, subsequently, the demineralization of teeth [42]. Periodontitis is considered to be caused by a multispecies community under the influence of keystone organisms with specific functions under environmental conditions that can disrupt homeostasis and cause dysbiosis and destructive inflammation [44]. Thus, it would make sense to control biofilms without altering the bacterial flora.

## 3. Intended Effects of Mouthwash on Biofilm Control

### 3.1. Study Selection and Data Collection

To reconfirm the intended effects of mouthwash on biofilm control, systematic reviews from the last decade were summarized. MEDLINE was searched for systematic reviews of randomized clinical trials (RCTs) and meta-analyses related to mouthwash published from 2012 to 20 March 2022. The search terms and results are summarized in Table 1. The titles and abstracts of the retrieved articles were reviewed and categorized according to the issues explored in this review.

**Figure 3:**
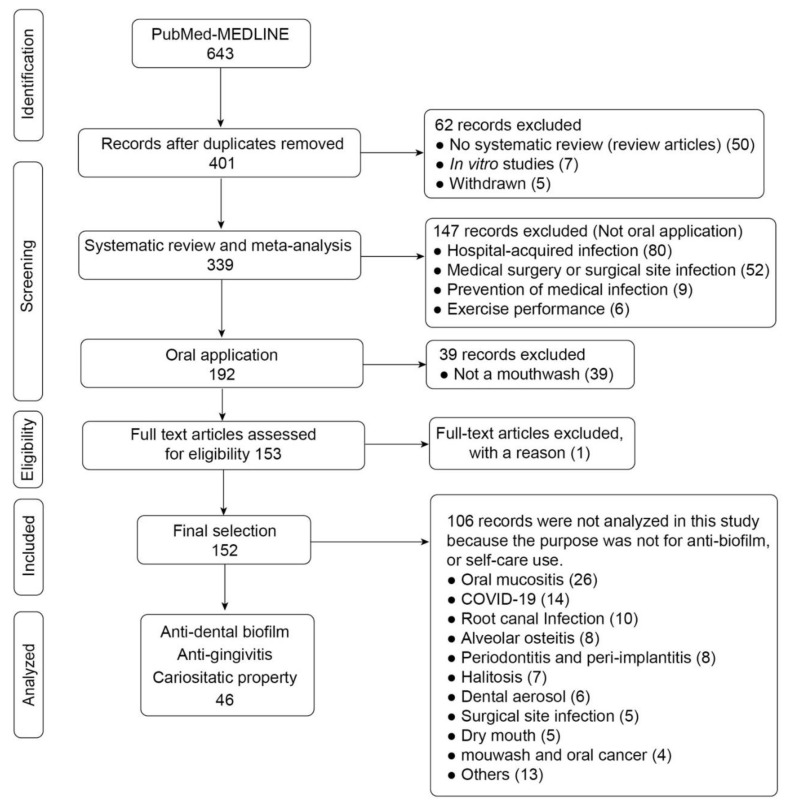
Flow diagram of the screening and selection process.

The inclusion criteria for the articles were as follows: (a) systematic review or meta-analysis of RCTs; (b) anti-biofilm effect; and (c) self-care use. Investigations funded by mouthwash manufacturers were not included. Single RCTs or meta-analyses using data from the same study group were also excluded. The flow diagram of the screening and selection process is provided in Figure 3.

### 3.2. Current Evidence on the Effects of Mouthwash

Twenty-eight systematic reviews and 18 meta-analyses that investigated the anti-biofilm effects of various types of mouthwash were analyzed. All the articles indicated the inhibitory effects of mouthwash using plaque indices (PI) and gingivitis indices (GI). The most commonly studied active agents were CHG and EO, followed by CPC, with a significant number of six-month RCTs on anti-dental biofilm and the anti-gingivitis effects of mouthwash. There was a consensus, which is supported by established guidelines [22].

Mouthwash is used to suppress bacterial adhesion and is not targeted at mature biofilms. Most of the clinical studies evaluated biofilm accumulation using a plaque index and gingivitis using a gingival index following professional mechanical tooth cleaning.

#### 3.2.1. CHG

CHG is a cationic bisbiguanide with a broad spectrum of antibacterial activity. CHG binds to almost every site in the mouth, including the teeth, mucosa, pellicle, and saliva, and exhibits antibacterial activity for up to 5 h [45]. Its mechanism of action is thought to be membrane disruption [45]. All the reviews had similar inclusion criteria, such as RCTs with at least four weeks of follow-up of daily use [16,46,47,48,49,50]. All the RCTs estimated the inhibition rate of the mouthwash used in conjunction with daily toothbrushing. A toothbrush and toothpaste were provided to all the participants to eliminate the effects of the dentifrice used. Five meta-analyses reported a reduction in PI and GI scores. The reduction rate calculated across the different reviews varied due to the different concentrations of CHG; despite the differences in the PI scores used and the follow-up periods, all the mouthwashes showed significant effects relative to the placebo rinse, even after 6 months without any professional care (Table 2 and Table 3). All the authors concluded that there was strong evidence in support of the efficacy of CHG as an anti-dental biofilm and anti-gingivitis agent. For example, the reduction in dental biofilm with a CHG mouthwash more than four weeks after the study period was 33% compared to the control [49]. Regarding gingivitis, an inhibition effect of up to 26% can be expected [49].

#### 3.2.2. EO

The EOs in the Listerine antiseptic were used to determine their long-term effects for more than 4 weeks. EO (Listerine) is an over-the-counter mouthwash containing a fixed formula of 0.064% thymol, 0.092% eucalyptol, 0.042% menthol, and 0.060% methyl salicylate. The mechanism of action of EO is membrane disruption at high concentrations and the inactivation of essential enzymes at lower concentrations [26]. Five meta-analyses reported reductions in PI [16,47,48,51,52], and five meta-analyses reported reductions in GI scores [16,48,50,51,52]. The PI reductions by EO after a follow-up of 6 months were similar among the different meta-analyses, unlike CHG, due to a fixed EO formula (Table 2). EO also significantly improved the PI and GI scores at six months with high reliability (Table 2 and Table 3). For example, there were weighted mean percentage reductions of 27% for PI and 18.2% for GI compared with the placebo group [53].

There was no consensus on the superiority of the EO and CHG mouthwashes for dental biofilm inhibition among the studies. Escribano et al. conducted mixed comparisons of the products and found no statistically significant difference between the CHG and EO mouthwashes (weighted mean difference (WMD) = −0.09, *p* = 0.58) [47]. By contrast, Van Leeuwen et al. reported that CHG had significantly better dental biofilm control effects than EO (WMD = 0.19, *p* = 0.0009) [25]. Boyle et al. reported that their reduction rates were significantly different, at 31.6% for CHG and 24% for EO, after three months, and quite similar, at 36% for CHG and 35% for EO, after six months [48].

Figuero et al. conducted a network meta-analysis and reported that non-alcoholic EO had the greatest effect on the GI scores from CHG (standard mean difference = 2.49, *p* < 0.05) and other active agents (2.25 to 3.38, *p* < 0.05) [50]. By contrast, van Leeuwen et al. reported, in a previous review, that no significant difference was found between EO and CHG in terms of the reduction in gingival inflammation [25]. Taken together, it seems more reasonable to choose a product that the patient can use rather than debate over which has the superior bactericidal activity.

#### 3.2.3. CPC

CPC is a cationic surface-active agent and has a broad antimicrobial spectrum. Its mechanism of antimicrobial activity involves the disruption of membrane function, followed by the leakage of cytoplasmic material and the collapse of the intra-cellular equilibrium [27]. Four meta-analyses reported reductions in PI scores [16,47,48,54], and four other meta-analyses reported reductions in GI scores [16,48,50,54]. CPC showed inconsistent results, especially at concentrations of less than 0.05% (Table 2 and Table 3). The most recent meta-analysis of the effect of CPC, as an adjunct to toothbrushing, on interproximal plaque and gingival inflammation was conducted by Langa et al. [54]. Studies with a minimum of 6 weeks of follow-up were included. Following an initial screening of 2,635 studies, 8 were selected. The CPC-based solutions used in all the studies significantly reduced the interproximal plaque scores. The authors concluded that CPC mouthwashes may be good alternatives for interproximal plaque removal, improving interproximal gingivitis.

A comparison of the PI reductions between CHG and CPC, and EO and CPC after 6 months, performed by Escribano et al., yielded WMDs of −0.37 (*p* = 0.03) and −0.46 (*p* = 0.00), respectively [47]. Similarly, Haps et al. conducted a systematic review to determine the efficacy of CPC mouthwashes and concluded that CPC provided a small, but significant, benefit for the control of dental biofilm [27]. Boyle et al. reported that CPC trials did not suggest a meaningful anti-gingivitis effect, and the relative reduction rates of CPC at three months and six months were 11.2% (95% confidence interval (Cl): −35.5 to +13.1) and 13.4% (95% Cl: −43.3 to +16.5), respectively [48]. CPC appeared to be less effective for the inhibition of dental biofilm and gingivitis than CHG and EO.

#### 3.2.4. Other Chemical Compounds

Some meta-analyses demonstrated the effects of mouthwashes including delmopinol [16,47,48,50], amine fluoride (AmF)/stannous fluoride (SnF) [16,31,50], alexidine [47,50], and triclosan [47,50]. The results were inconsistent, and their effects were not significantly different from those of placebo rinse, except for triclosan (Table 2 and Table 3). It is not possible, at this time, to conclude as to the effectiveness of these mouthwashes for the control of subgingival biofilms.

#### 3.2.5. Natural Products

CHG mouthwashes have superior antibiofilm properties; however, their long-term use causes undesirable effects, including altered taste perception, the staining of teeth and tongue, and burning sensations [55,56]. The interest in natural compounds is therefore inceasing, as they are considered safe for living organisms [34]. The natural compounds used in mouthwashes include *Azadirachta indica* (neem), *Camellia sinensis*, *Calendula officinalis*, *Elettaria cardamomum* (ela), *Ixora coccinea* Linn., *Leptospermum scoparium*, *Melaleuca alternifolia*, *Rosmarinus Officinalis* L., *Sesamum indicum*, and *Zingiber officinale* [57,58,59,60,61,62,63]. The biofilm reduction rates of natural compounds have been compared with those of placebo and the CHG mouthwash. Most meta-analyses have demonstrated that natural compounds and the CHG mouthwash have comparable efficacy in reducing dental biofilm and gingival inflammation (Table 2 and Table 3). However, most of these meta-analyses had serious limitations, such as small samples, ununified durations, and moderate (I^2^ = 40–80%) or considerable (>80%) heterogeneity, resulting in moderate-to-low quality of evidence. Thus, no conclusions can be reached, at this time, as to the effectiveness of natural products.

For example, curcumin is a yellow polyphenolic pigment from the *Curcuma longa* L. (turmeric) rhizome that has been used for culinary and food-coloring purposes [64]. It has been reported that curcumin exhibits several biological activities, including antioxidant, antimicrobial, anti-inflammatory, hepatoprotective, cardioprotective, antirheumatic, neuroprotective, and anticancer properties [64]. Al-Maweri et al. reported a meta-analysis comparing the anti-dental biofilm and anti-gingival inflammation effects of curcumin and CHX [60]. Of 210 articles, 6 publications met the criteria for inclusion. The efficacies of the curcumin and CHG mouthwashes were found to be comparable (Table 2 and Table 3). However, the authors also suggested that further multicenter clinical trials with standardized methodologies and an adequate evaluation period were needed, because five out of the six studies were performed in India and the samples of three out of the six studies included only ten participants.

More recently, Aljameel et al. reported a systematic review and meta-analysis of the effects of Triphala mouthwashes on plaque and gingival reductions [65]. Triphala comprises a distinct combination of fruits from three medicinal trees, namely Amalaki (*Embalica officinalis*), Vibhitaki (*Terminalia belerica*), and Haritaki (*Terminalia chebula*) [66]. Seven RCTs were included in the analysis, and the efficacy of Triphala was compared with that of 0.1–0.2% CHG. The results showed that the Triphala mouthwashes improved the clinical gingival inflammatory parameters of plaque-induced gingivitis with equal clinical efficacy to CHG mouthwash. However, the durations of the follow-ups of the studies varied from 2 weeks to 63 weeks, and it seems that more tests within the same period are needed. Altogether, mouthwashes containing natural compounds may be recommended as substitutes for more conventional over-the-counter oral hygiene products; however, the quality of evidence in support of their use appears to be low at this time.

#### 3.2.6. Sodium Hypochlorite (NaOCl)

NaOCl is an excellent non-specific proteolytic and antimicrobial agent. Its solution has been used as the gold standard for disinfecting entire root-canal systems [67]. Hussain et al. conducted a systematic review of the effect of a sodium hypochlorite mouthwash on biofilm reduction and periodontic parameters [32]. NaOCl at concentrations between 0.05 and 0.25% was used. After a review of 833 titles, 7 eligible papers were retrieved. The authors did not conduct a meta-analysis because the studies showed considerable heterogeneity in terms of their methodological and clinical features. In addition, NaOCl had some non-negligible side effects, including the unpleasant taste of bleach, altered taste, extrinsic brown tooth stains, redness of the tongue, and a burning sensation [32].

#### 3.2.7. Chlorine Dioxide (ClO_2_)

One meta-analysis and one systematic review assessed the efficacy of ClO_2_ [33]. ClO_2_ is an oxidizing biocide with a powerful bactericidal property, which kills microorganisms by disrupting the transport of nutrients across the cell wall [68]. Kerémi et al. conducted a systematic review and meta-analysis of the effects of ClO_2_ on oral hygiene [33]. Only 5 out of 364 articles were eligible. The results showed that ClO_2_ reduced both plaque and gingivitis indices and bacterial counts in the oral cavity in a similar way to routinely used oral rinses, including CHG and herbal mouthwashes. However, the authors stated that the evidence supporting the outcome was very limited because of the varied study durations and designs. Santos et al. used a systematic review to determine the antimicrobial effects of ClO_2_ mouthwashes [31]. Five articles compared the efficacies of ClO_2_ and CHG. The antimicrobial effects of ClO_2_ were even greater than those of CHG. However, the concentrations of the solutions used in the studies were different. Furthermore, the authors highlighted the heterogeneity of the studies as one of the limitations, and no meta-analysis was conducted.

#### 3.2.8. Cariostatic Property: Children and Adolescents

The benefits of fluoride for carie prevention and arrest are generally accepted by dentists, and fluoride has been widely used in various applications, including water fluoridation, toothpaste, gel, varnish, and rinse, depending on the setting, such as public places, dental offices, and homes [4]. Fluoride mouth rinses have been used under supervision in school-based programs to prevent tooth decay [69]. Mouth rinses are also recommended, especially for patients who have a high risk of caries due to radiation exposure, hyposalivation, xerostomia, the presence of difficult brushing sites, such as interproximal root surfaces, and orthodontic treatment [70]. Although extensive epidemiological studies have shown the effectiveness of fluoride mouthrinses, especially for children, systematic reviews and meta-analyses have not been conducted in recent years.

There are two systematic reviews on this issue. Marinho et al. conducted one of these systematic reviews to evaluate the efficacy of fluoride mouthrinses in preventing dental caries in children and adolescents up to 16 years of age [69]. Studies lasting for at least one year were included. The main outcome was the worsening of caries, as indicated by changes in the decayed, missing, and filled tooth surfaces (DMFS) of permanent teeth. TIn total, 35 trials, involving 15,305 children and adolescents, met the inclusion criteria. The effects of fluoride mouthwash alone without any intervention with caries-preventive agents, such as fluoridated dentifrice, CHG, sealants, and xylitol chewing gum, were analyzed. The results showed that the regular and supervised use of fluoride mouthrinse was associated with a remarkable slowing-down of caries progression, showing a 27% decrease in the DMFS index (95% Cl: 23–30%) and a 23% decrease in the decayed, missing, and filled-teeth (DMFT) index (95% Cl: 18–29%). No acute adverse symptoms were reported in any of the trials.

Although most people use a high-concentration fluoridated dentifrice [70], it is not clear whether combining a fluoride dentifrice with a mouthrinse has an additional effect. In another systematic review conducted by Marinho et al., the size of the effect of a fluoride mouthrinse combined with a fluoride toothpaste was compared to that of toothpaste alone [71]. The fraction pooled estimate of the random-effects meta-analysis of five trials (*n* = 2738) on the preventive effects on DMFS was only 0.07 (95% Cl: 0.0–0.13), with no significant difference (*p* = 0.06). Another systematic review, by Twetman et al., concluded that a sodium fluoride mouthrinse may have an anti-caries effect in children with limited fluoride exposure, while its additional effect in children when combined with the daily use of fluoride toothpaste is questionable [72].

Fluoride mouthwashes with various active ingredients, such as sodium fluoride, stannous (Sn) fluoride, and sodium monofluorophosphate, are available [73]. In terms of the additional effect, it was not possible to conclude as to which of the components of fluoride mouthrinses had the best preventive effects. More recently, Zanatta et al. conducted a systematic review and meta-analysis to evaluate the effects of different fluoride types and vehicles on the prevention of enamel erosion and erosive tooth-wear progression [74]. Of 318 articles, 13 were selected to compare the ingredients of different mouthrinses. Under erosive/abrasive challenges, the fluoride solutions had different inhibitory effects on enamel loss. The Sn formulations were the most effective (−11,49; 95% Cl: −16.62 to −6.37); however, sodium fluoride (NaF) showed no significant effect (−2.83; 95% Cl: −8.04 to 2.38). These results indicated that the stannous-enriched fluoride offered the highest protection against enamel erosion and erosive wear. 

#### 3.2.9. Cariostatic Property: Root Caries

Two systematic reviews investigated the preventive effects of mouthwash against dental-root caries.

Zhang et al. evaluated the effects of topical fluoride on the prevention of dental-root caries using a network meta-analysis [75]. Clinical visual and tactile assessments were used for the diagnosis of root caries in this review. The results showed that the daily use of a 0.2% sodium fluoride (NaF) mouthrinse was more effective than placebo (mean difference (MD) = −1.90, 95% CI= −3.48 to −0.32). Moreover, the use of a 0.2% NaF mouthrinse was significantly more effective at reducing root caries than a 0.05% fluoride mouthrinse (MD = −1.78, 95% CI = −3.37 to −0.20). The evidence for the preventive effect of a 0.05% fluoride mouthrinse relative to placebo was unclear (MD = −0.12, 95% CI = −0.29 to 0.06). The authors concluded that a 0.2% NaF mouthrinse is likely to be the most effective, followed by a combination of 1100–1500-parts-per-million fluoride toothpaste with a 0.05% NaF mouthrinse, and a 1100–1500-part-per-million fluoride toothpaste only. However, the level of evidence was considered low due to the small number of studies included, the risk of bias, and imprecision. Moreover, the possible confounding factors were not considered. The authors mentioned the necessity of further studies for people at high risk.

Wierichs et al. reviewed the clinical studies investigating chemical agents for the prevention or inactivation of root caries [76]. The outcomes were evaluated using the root caries index (RCI) and decayed, missing, filled-root surfaces (DMFRS)/DFRS. The mouthrinses containing 225–900 ppm of fluoride showed a more significant reduction in DMFRS/DFRS than a placebo rinse (MD = −0.18, 95% CI = −0.35 to −0.01). However, the inhibition effect was much lower than that of 38% silver diamine fluoride (SDF) (MD = −0.33; 95% Cl = −0.39 to −0.28). The authors concluded that these results should be interpreted carefully due to the low number of clinical trials, high risk of bias within the studies, and limited grade of evidence.

Schwendicke et al. performed a simulation based on recent data from randomized controlled trials and systematic reviews to determine cost-effective management strategies for root carie lesions [77]. In their study, patients were simulated over 10 years using a Markov model, and the following four treatments were evaluated: no treatment, daily 225–800-parts-per-million fluoride rinses, chlorhexidine varnish, and silver diamine fluoride varnish. The results of this simulation study indicated that fluoride rinses were accepted for their cost-effectiveness if the willingness-to-pay threshold increased, but this acceptability never exceeded 40%. The authors concluded that fluoride rinses are not cost-effective for populations with more teeth and high tooth-level risks, and the application of SDF was recommended as a cost-saving treatment for the prevention of root caries in patients with a high risk of developing root caries.

## 4. Side Effects and Potential Adverse Reactions to Mouthwashes

Although the current evidence, based on systematic reviews, supports the efficacy of various active ingredients in therapeutic mouthwashes for preventing biofilm accumulation and gingivitis, the various side effects of these mouthwashes should be considered. Furthermore, some potential adverse reactions to biofilm control through mouthwashes have recently been reported.

### 4.1. Side Effects (Patients’ Complaints)

The long-term use, for more than 4 weeks, of CHG mouthwash causes a large increase in extrinsic tooth staining [46]. Various other side effects of CHG mouthwash have also been reported, including the following: taste disturbances/alterations; effects on the oral mucosa, including soreness, irritation, mild desquamation, and mucosal ulceration/erosions; a general burning sensation, or a burning tongue; and the promotion of calculus [46,62,63]. A loss of taste and numbness were significantly more frequent with 0.12 and 0.2% CHG than with 0.06% CHG [78]. CHG is therefore restricted to short-term or moderate-term use and to special clinical situations [53].

Poor taste [79], tooth staining, calculus, taste alteration, and burning sensation at the oral mucosa, tongue-tip, and gingiva have been reported by some RCTs as common side effects of mouthwashes containing EO and alcohol as solvent [80,81]. Althouth EO without alcohol is now commercially available, its effects on taste perception have not significantly improved [82,83]. The acceptability of EO mouthwashes depends on patients because, in some other studies, no or few patients reported side effects [84,85].

CPC mouthwash can cause similar side effects, including staining, burning, taste alterations, mouth ulcers, dysgeusia, and glossodynia [81]. Although herb-based mouthwashes revealed better effectiveness with fewer side effects, no conclusions can be reached as to the effectiveness of their anti-biofilm property at this time [86]. As mouthwashes are effective when used daily, patients’ impressions of mouthwash products after trying them are very important.

### 4.2. Cytotoxic Effects on Human Cells

There are some in vitro studies demonstrating that mouthwashes, including CHG [87,88,89,90], hydrogen peroxide [87,89], CPC [87,88], ClO_2_ [87], EO [87,89,90], and povidone-iodine [87,89], have concentration-dependent cytotoxic effects on human gingival epithelial cells. All these studies showed that all the above rinses were highly toxic when used undiluted. CHG should be diluted to at least 6.3% [90] and EO to 10% [87] to attain non-toxic concentrations.

### 4.3. Antimicrobial Resistance

One emerging issue with strategies that rely on eradication is antimicrobial resistance, whereby microorganisms reduce the effectiveness of mouthwashes [91]. This phenomenon is likely to occur, but it has not been focused on thus far. Recent investigations raised an alarm regarding the long-term use of CHG due to the risk of the emergence of antimicrobial-resistant bacteria through increased efflux pump activity and cell membrane change [92,93]. Muehler et al. investigated the transcriptomic stress response in *S. mutans* after treatment with CHG using RNA sequencing. An analysis of differential gene expression following a pathway analysis revealed a considerable number of genes and pathways significantly up- or downregulated after sublethal treatment with CHG, showing the involvement of gene regulation in purine nucleotide synthesis, biofilm formation, transport systems, and stress responses [94]. However, it remains unclear whether persister cells in oral biofilms, which have frequently been treated with CHG, and resistant strains contribute to the development of cross-resistance in oral biofilms in vivo. 

### 4.4. Residual Structure

Mouthwash is effective in suppressing the adhesion of oral bacteria, but it is less effevtive against mature biofilms. It has been reported that almost all mouthwashes failed to remove biofilms even if the constituent microorganisms were completely eliminated. Studies have shown that little or no biofilm was removed when in vitro oral biofilms were treated with ethanol [95,96], CHG [95,96,97,98], nisin [96,99], glutaraldehyde [99], a quaternary ammonium compound [99], sodium lauryl sulfate [96], triclosan [96], CPC [96,97], and EO [95]. Using bacterial vibration spectroscopy and attenuated-total-reflectance–Fourier-transform infrared spectroscopy, Song et al. demonstrated that oral bacteria adhering to salivary conditioning films became more difficult to remove after exposure to mouthwashes containing CHG, CPC, and AmF due to the strengthening of the polysaccharide bond [100].

The residual structure may serve as a scaffold for biofilm re-development [101]. In an in vitro study using a rotating disc reactor and the confocal laser-scanning microscopic observation of longitudinal cryosectioned biofilms, Ohsumi et al. demonstrated that a disinfected *Streptococcus mutans* biofilm structure favored secondary bacterial adhesion. The structure may also act as a source of antigens, which induce host inflammatory reactions because extracellular polymeric substances contain carbohydrates, proteins, polysaccharides, lipids, and nucleic acids [102]. For example, the lipid A moiety of lipopolysaccharides initiates innate immune responses by interacting with Toll-like receptor 4 [103].

In addition, the residual biofilm structure absorbs calcium and phosphate from saliva and/or crevicular fluid, resulting in calculus formation. Although the calculus surface may not, in itself, induce inflammation in the adjacent periodontal tissue, it is known to cause plaque retention [104].

### 4.5. Retarded Penetration into Biofilm and Promotion of Biofilm Development

It has been demonstrated that the penetration of antimicrobial compounds in biofilms is low, as is that of mouthwashes, especially during short-term exposure. Direct time-lapse microscopy revealed that the penetration of an oral biofilm model by 0.12% CHG was critically restricted, indicating that the average penetration velocity was only 4.1 µm/min [95]. Wakamatsu et al. reported the kinetics of the penetration of in vitro *S. mutans* biofilms by mouthwashes using direct time-lapse microscopy [97]. All the mouthwashes demonstrated retarded penetration, with penetration velocities ranging from 4.2 to 30.1 µm/min.

The retarded penetration of mouthwash can cause the exposure of the microorganisms inside the biofilm to sub-minimum inhibitory concentrations (sub-MICs) of active ingredients, and this antimicrobial stress may upregulate pathogenic genes and facilitate horizontal gene transfer [105]. Kaplan et al. demonstrated that the biofilm formation of *Staphylococcus aureus* significantly increased in the presence of four different β-lactam antibiotics at sub-MIC [106]. The amount of biofilm induction was 10-fold at its maximum, and sub-MIC β-lactam antibiotics induced autolysin-dependent extracellular DNA release. Furhtermore, for oral biofilms, there are some studies reporting that the sub-MICs of antimicrobial agents upregulated the genes related to pathogenicity. Suzuki et al. investigated the effects of sub-MIC CHG on the development of in vitro multi-species biofilms [107]. The results indicated that CHG at a specific sub-MIC enhanced gene transcription related to biofilm formation and promoted the development of a multi-species biofilm. Representative expmeriments demonstrating the upregulation of pathogenic genes at the sub-MIC are summarized in Table 4 [108,109,110]. However, since these experiments were performed in vitro, it is not clear whether the enhanced biofilm formation observed is clinically significant.

### 4.6. Ecological Changes in the Oral Biofilm Microbiota

Oral diseases, such as dental caries and periodontitis, develop when there is an imbalance in the oral microbiome. It has been suggested that wide-spectrum antimicrobials, such as CHG and EO mouthwashes, kill all bacteria non-selectively and do not change the bacterial flora. However, the appearance of resistant bacteria and decrease in drug sensitivity can cause dysbiosis, contribute to oral infection, and exert potential adverse effects on systemic conditions.

Since there are mixed findings on the effect of mouthwash on the biofilm bacterial composition in the biofilm [111,112,113,114], no consensus has yet been established. However, recent studies analyzing the biofilm microbiome through amplicon sequencing have reported interesting findings. 

Bescos et al. investigated the effect of the seven-day use of CHG mouthwash on the salivary microbiome, as well as on several saliva and plasma biomarkers, in 36 healthy individuals. The results showed that the CHG mouthwash significantly increased the Firmicute and Proteobacteria levels, leading to more acidic conditions and lower nitrite availability in the healthy individuals [115]. Mostajo et al. investigated the influence of three different mouthwashes on the bacterial composition and metabolic activity of oral biofilms in vitro [111]. All the studied mouthwashes affected the in vitro biofilm differently. The microbial diversity was reduced following treatment with 0.12% CHG mouthwash. The oxygenating agent treatment caused significant changes in the bacterial composition, with lower proportions of Veillonella and higher proportions of non-mutans, compared to the control. Chatzigiannidou et al. reported that 0.12% CHG treatment induced profound shifts in microbiota composition and metabolic activity using two types of oral biofilm model [112]. The authors concluded that there is a need for alternative treatments that selectively target the disease-associated bacteria in the biofilm without targeting commensal microorganisms. It remains unclear whether these ecological changes to the microbiota caused by mouthwash have clinically significant adverse effects. Further in vivo experiments are required to elucidate whether mouthwashes cause dysbiosis, which adversely affects oral health. 

## 5. Conclusions

**Figure 4:**
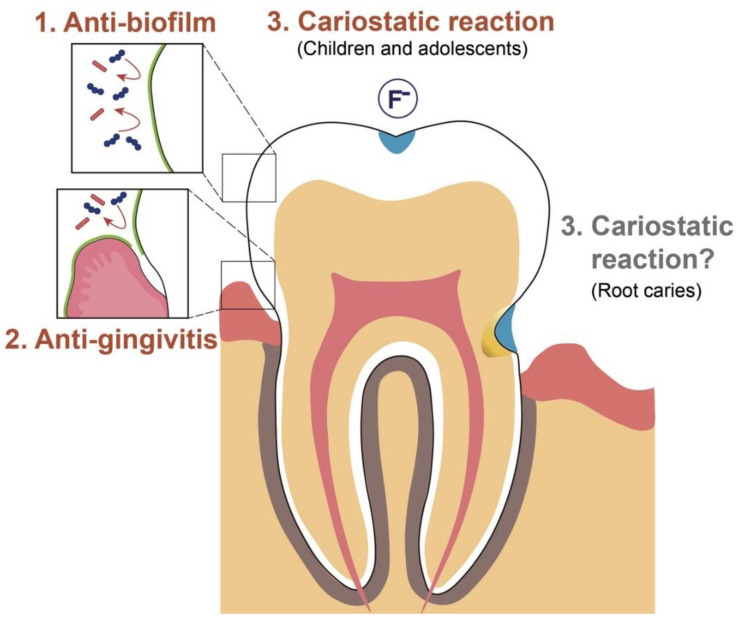
Schematic diagram of the clinical effects of mouthwashes against supragingival dental biofilm with strong evidence. The anti-biofilm property (1) has been proven to be effective, followed by the anti-gingivitis property (2), and their cariostatic actions are aimed at children and adolescents (3). There are no conclusive findings regarding the preventive effect of fluoride mouthwash alone on root caries.

Mouthwash is used for rinsing and can reduce the bacterial load of the entire mouth. It is intended to suppress bacterial adhesion during biofilm formation processes and is not targeted at mature biofilms. The most common evidence-based effects of mouthwash on subgingival biofilms include the inhibition of biofilm accumulation and their anti-gingivitis effect. There has been no significant change in the strength of the evidence over the last decade. There is strong evidence for the effects of CHG and EO mouthwash. Fluoride mouthwash contributes to caries prevention and arrest, with high-quality evidence for its effectiveness in children and limited evidence for its role in root-caries management (Figure 4).

**Figure 5:**
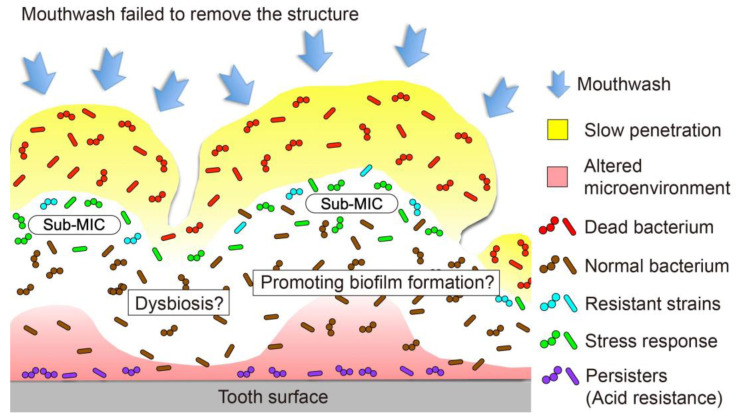
Posible adverse reactions inside the mature oral biofilm. Exposure of a mature oral biofilm to mouthwash is associated with several possible adverse reactions, such as the emergence of a resistant strain, effects of the residual structure, enhanced pathogenicity following retarded penetration, and ecological changes to the microbiota.

The exposure of mature oral biofilms to mouthwash is associated with several possible adverse reactions, such as the emergence of resistant strains, the effects of the residual structure, enhanced pathogenicity following retarded penetration, and ecological changes to the microbiota (Figure 5). These concerns require further elucidation. In future, the strategy for oral biofilm control may shift to reducing the biofilm through detachment or by modulating its quality, rather than eliminating it, to preserve the benefits of the normal resident oral microflora.

## Figures and Tables

**Table 1:** A summary of search terms and results in this study.

Sequence No.	Terms and Strategy (Publication Dates from 2012 to 2022)	Hits
#1	(“mouthwashes” [Pharmacological Action] OR “mouthwashes” [MeSH Terms] OR “mouthwashes” [All Fields] OR “mouthwash” [All Fields] OR “mouthwashing” [All Fields] OR “mouthwashings” [All Fields]) AND (“systematic review” [Publication Type] OR “systematic reviews as topic” [MeSH Terms] OR “systematic review” [All Fields])	300
#2	(“mouthwashes” [Pharmacological Action] OR “mouthwashes” [MeSH Terms] OR “mouthwashes” [All Fields] OR “mouthwash” [All Fields] OR “mouthwashing” [All Fields] OR “mouthwashings” [All Fields]) AND (“meta analysis” [Publication Type] OR “meta analysis as topic” [MeSH Terms] OR “meta analysis” [All Fields])	246
#3	(“mouthrinse” [All Fields] OR “mouthrinsed” [All Fields] OR “mouthrinses” [All Fields] OR “mouthrinsing” [All Fields] OR “mouthrinsings” [All Fields]) AND (“systematic review” [Publication Type] OR “systematic reviews as topic” [MeSH Terms] OR “systematic review” [All Fields])	57
#4	(“mouthrinse” [All Fields] OR “mouthrinsed” [All Fields] OR “mouthrinses” [All Fields] OR “mouthrinsing” [All Fields] OR “mouthrinsings” [All Fields]) AND (“meta analysis” [Publication Type] OR “meta analysis as topic” [MeSH Terms] OR “meta analysis” [All Fields])	40

**Table 2:** Summary of the reductions in plaque indices.

Solutions	*n*	Weighted Mean Difference (95% Cl)	Index	Compared Control	Follow-up Periods	Reference
CHG	12	−1.45 (−1.00 to −1.90)	QHI	Baseline	4 to 6 weeks	[46]
	4	−0.78 (−1.07 to −0.49)	TQHI	Placebo	6 months	[47]
	3	−0.640 (−0.756 to −0.524)	TQHI	Placebo	6 months	[16]
	2	−0.208 (−0.351 to −0.065)	PI	Placebo	6 months	[16]
	17	−0.362 (−0.571 to −0.153) ^†^	QHI or TQHI	Baseline	≥ 4 weeks	[48]
	5	−0.39 (−0.70 to −0.08)	PI	Baseline	6w or 6m	[49]
	10	−0.67 (−0.82 to −0.52)	QHI	Baseline	4w to 6m	[49]
EO	9	−0.86 (−1.05 to −0.68)	TQHI	Placebo	6 months	[47]
	9	−0.827 (−1.053 to −0.600)	TQHI	Placebo	6 months	[16]
	16	−0.265 (−0.405 to −0.124) ^†^	QHI or TQHI	Baseline	≥ 4 weeks	[48]
	14	−0.86 (−1.05 to −0.66)	QHI	Placebo	6 months	[51]
	4	−0.39 (−0.3 to −0.47)	QHI	21.6 or 26.9% hydro-alcohol	6 months	[52]
CPC (>0.05%)	6	−0.41 (−0.65 to −0.17)	TQHI	Placebo	6 months	[47]
	3	−0.465 (−0.631 to −0.299)	TQHI	Placebo	6 months	[16]
	8	−0.112 (−0.273 to 0.029) *^,†^	TQHI	Baseline	≥ 4 weeks	[48]
	9	−0.70 (−0.83 to −0.57)	PI, TQHI, MPI	Placebo	≥ 6 weeks	[54]
CPC (<0.05%)	3	−0.26 (0.07 to −0.55) *	TQHI	Placebo	6 months	[47]
Del	2	−0.24 (−0.67 to 0.19) *	TQHI	Placebo	6 months	[47]
	3	−0.144 (−0.231 to −0.058)	TQHI	Placebo	6 months	[16]
	4	−0.173 (−0.853 to 0.507) *^,†^	TQHI	Baseline	4 weeks	[48]
AmF/SnF	2	−0.079 (−0.260 to 0.101) *	TQHI	Placebo	6 months	[31]
	2	−0.195 (−0.335 to −0.054)	PI	Placebo	6 months	[31]
Alexi	2	−0.18 (−0.60 to 0.24) *	TQHI	Placebo	6 months	[47]
Tric	3	−0.67 (−1.05 to −0.30)	TQHI	Placebo	6 months	[47]
Herb	6	−2.93 (−6.43 to 0.58) *	PI or TQHI	CHG	4 weeks	[57]
	6	2.61 (4.42 to 0.9)	PI or TQHI	CHG or Placebo	12 weeks	[57]
	11	0.22 (0.20 to 0.24)	PI or QHI or TQHI	CHG		[58]
	5	−0.61 (−0.80 to −0.42)	QHI	Placebo	2w to 3 M	[59]
	5	0.08 (−0.19 to 0.34) *	PI	CHG	10d to 3w	[59]
	5	0.00 (−0.04 to 0.04) *	TQHI	CHG	2 or 4w	[59]
Curcumin	6	0.27 (−0.53 to 1.07) *	PI or TQHI	CHG	21 or 28 days	[60]
Propolis	3	−1.24 (−2.51 to 0.04) *	PI	Placebo	3 or 5 days	[61]
*Salvadora persica*	12	−0.46 (−0.29 to −0.63)	PI or TQHI	Placebo	4d to 2m	[62]
*Salvadora persica*	18	0.19 (0.01 to 0.37) *	PI or TQHI	CHG	4d to 2m	[62]
Green Tea	5	−0.14(−1.80 to 1.43) *	PI	CHG	2 to 4w	[63]
ClO_2_	5	−0.720 (−0.487 to −0.952)	PI	Placebo	7 days to 5w	[33]

*n*: number of studies, CHG: chlorhexidine gluconate mouthwash, EO: essential-oil-containing mouthwash (Listerine antiseptic), CPC: cetylpyridinium chloride mouthwash, Del: delmopinol mouthwash, AmF: amine fluoride mouthwash, SnF: stannous fluoride mouthwash, Alexi: alexidine, Tric: triclosan, PI: plaque index (Loe and Silness), QHI: Quigley-Hein plaque index, TQHI: Turesky modification of the Quigley-Hein plaque index, MPI: modified proximal index. ^†^ Expressed as the summary relative difference meaning a percentage change from the baseline. * No significant difference (*p* > 0.05).

**Table 3:** Summary for the reduction of gingival indices.

Solutions	*n*	Weighted Mean Difference (95% Cl)	Index	Compared Control	Follow-up Periods	Reference
CHG	3	−1.20 (−0.23 to −2.16)	GI or MGI	Placebo	6 months	[50]
	10	−0.21 (−0.11 to −0.31)	GI	Baseline	4 to 6 weeks	[46]
	4	−0.185 (−0.285 to −0.086)	GI	Placebo	6 months	[16]
	19	−0.223 (−0.412 to −0.034) ^†^	GI or MGI	Baseline	≥ 4 weeks	[48]
	8	−0.32 (−0.42 to −0.23)	GI	Baseline	6w, 3m, 6m	[49]
EO	9	−1.44 (−0.82 to −2.06)	GI or MGI	Placebo	6 months	[50]
	2	−0.133 (−0.194 to −0.072)	GI	Placebo	6 months	[16]
	8	−0.537 (−0.764 to −0.311)	MGI	Placebo	6 months	[16]
	16	−0.203 (−0.312 to −0.093) ^†^	GI or MGI	Baseline	≥ 4 weeks	[48]
	2	−0.36 (−0.26 to −0.62)	GI	21.6 or 26.9% hydro-alcohol	6 months	[52]
	11	−0.52 (−0.67 to −0.37)	MGI	Placebo	6 months	[51]
	3	−0.24 (−0.46 to −0.01)	GI	Placebo	6 months	[51]
CPC (>0.05%)	2	−1.04 (0.06 to −2.14) *	GI or MGI	Placebo	6 months	[50]
	5	−0.70 (−0.83 to −0.57)	GI	Placebo	≥ 6 weeks	[54]
	3	−0.344 (−0.627 to −0.062)	GI	Placebo	6 months	[16]
	2	−0.357 (−0.483 to −0.231)	MGI	Placebo	6 months	[16]
	8	−0.126 (−0.312 to 0.059) *^,†^	GI or MGI	Baseline	≥ 4 weeks	[48]
CPC (≤0.05%)	3	−0.31 (0.76 to −1.38) *	GI or MGI	Placebo	6 months	[50]
Del	1	−0.06 (1.86 to −1.98) *	GI or MGI	Placebo	6 months	[50]
	2	−0.038 (−0.145 to −0.069) *	MGI	Placebo	6 months	[16]
	3	−0.014 (−2.337 to 2.308) ^†,^*	GI or MGI	Baseline	≥ 4 weeks	[48]
AmF/SnF AmF/SnF	1	−0.76 (0.72 to −2.25) *	GI or MGI	Placebo	6 months	[50]
	2	−0.248 (−0.427 to −0.069)	GI	Placebo	6 months	[16]
Alexi	1	−0.16 (1.77 to −2.09) *	GI or MGI	Placebo	6 months	[50]
Tric	3	−1.50 (−0.36 to −2.62)	GI or MGI	Placebo	6 months	[50]
Herb	6	−0.15 (−0.32 to 0.01) *	GI	CHG	4 weeks	[57]
	5	−0.09 (−0.25 to 0.08) *	GI	CHG	12 weeks	[57]
	3	−0.28 (−0.51 to −0.06)	GI	Placebo	3 w or 3 m	[59]
	3	−0.59 (−1.08 to −0.11)	MGI	Placebo	2 to 4 weeks	[59]
	5	−0.07 (−0.22 to 0.07) *	GI	CHG	2 to 4 w	[59]
Curcumin	6	−0.13 (−0.35 to 0.09) *	GI	CHG	21 or 28 days	[60]
Green Tea	5	0.43(−0.63 to 1.49) *	GI	CHG	2 to 4 w	[63]
ClO_2_	4	−0.712 (−0.457 to −0.967)	GI	Placebo	7 to 21 days	[33]

*n*: number of studies, CHG: chlorhexidine gluconate, EO: essential oils (Listerine antiseptic), CPC: cetylpyridinium chloride. Del: delmopinol, AmF/SnF: amine fluoride/stannous fluoride, Alexi: alexidine, Tric: triclosan, GI: gingival index (Löe and Silness), MGI: modified gingival index. ^†^ Expressed as the summary relative difference, meaning a percentage change from the baseline. * There was no significant difference (*p* > 0.05).

**Table 4:** A summary of representative experiments demonstrating that sub-minimum inhibitory concentration of antimicrobial agents upregulated pathogenic genes.

Biocide	Concentration	Species	Condition of Bacteria	Incubation Time	Upregulated Genes	Reference
NaF, CHG, Tea polyphenol	1/2 MIC	*S. mutans*	Planktonic	24 h	*gtfB, gtfC, luxS, comD, comE*	[108]
NaF, CHG, Tea polyphenol	1/2 MIC	*S. mutans*	Biofilm	24 h	*gtfB, gtfC, luxS, comD, comE*	[108]
Triclosan	1/2 and 1/4 MIC	*S. mutans*	Planktonic	2 h	*atlA, gtfB, gtfC, comD, luxS*	[109]
MTAD, MTADN, MTAN	1/4 MIC	*Porphyromonas gingivalis*	Planktonic	1 h	*clpC, clpP* (MTAD, MTADN, MTAN), *sprE* (MTAD, MTADN), *ace, clpX, cylB, efaA, gelE* (MTAN)	[110]
CHG	1/10 MIC	*S. mutans*, *Streptococcus oralis, Actinomyces naeslundii*	Biofilm	48 h	*gtfB, gtfC, gtfD, comD, luxS*	[107]

MTAD: 3% doxycycline, 4.5% citric acid, and 0.5% polysorbate 80 detergent. MTADN: nisin combined with MTAD. MTAN: nisin in place of doxycycline in MTAD.

## Data Availability

The data sets used during the study are available from the corresponding author on reasonable request.
